# Supplementary material for: Asphalt Incorporation with Ethylene Vinyl Acetate (EVA) Copolymer and Natural Rubber (NR) Thermoplastic Vulcanizates (TPVs): Effects of TPV Gel Content on Physical and Rheological Properties
Source: Polymers (Basel). 2021 Apr 26;13(9):1397. doi: 10.3390/polym13091397 (PMC8123437; doi:10.3390/polym13091397)
Supplement: Supplementary file 1 [file polymers-13-01397-s001.zip › polymers-1146170-supplementary.pdf]

polymers

MDPI

Article

Asphalt incorporation with ethylene vinyl acetate copolymer and natural rubber thermoplastic vulcanizates (TPV): Effect of TPV gel content on physical and rheological properties

Nappaphan Kunanusont [1], Boonchai Sangpetngam [2] and Anongnat Somwangthanaroj [1,3,*]

[1] Department of Chemical Engineering, Faculty of Engineering, Chulalongkorn University, Bangkok 10330, Thailand (N.K. mai2newwood@hotmail.com)
[2] Department of Civil Engineering, Faculty of Engineering, Chulalongkorn University, Bangkok 10330, Thailand (B.S. boonchai.sa@chula.ac.th)
[3] Bio-Circular-Green economy Technology & Engineering Center, BCGeTEC, Faculty of Engineering, Chulalongkorn University, Bangkok 10330, Thailand
* Correspondence: anongnat.s@chula.ac.th

Supplement materials

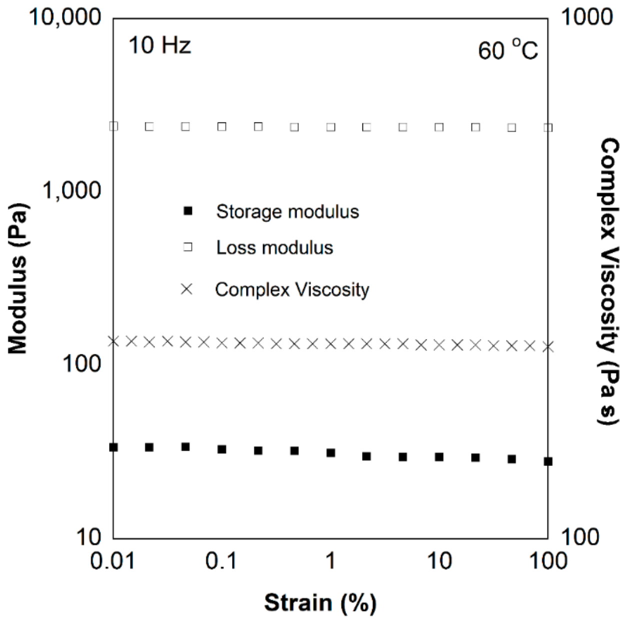

Figure S1. Rheological properties as a function of strain of neat asphalt at 60 °C.

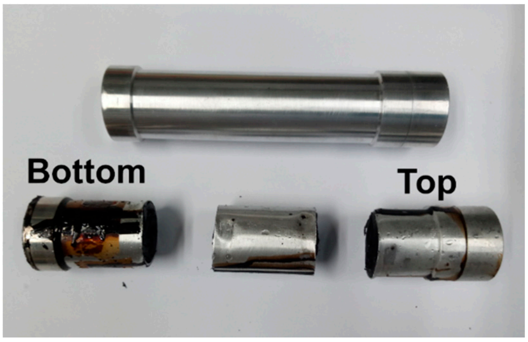

Figure S2. Appearance of aluminum tube and its three section: Top, Middle, and Bottom. The softening temperature of PMA from top and bottom section was evaluated.

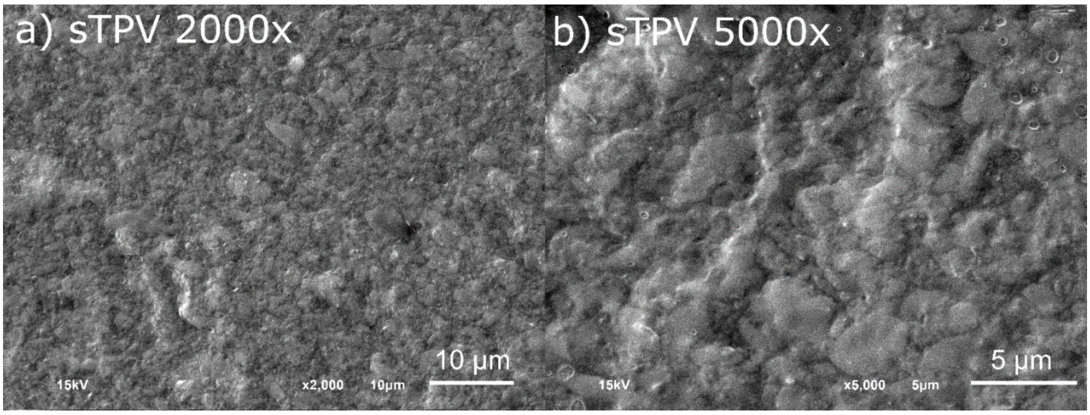

Figure S3. Fractured surfaces of EVA/NR TPVs with magnification of 2000 and 5000 [1].

Table S1. Specification of asphalt cement, natural rubber modified asphalt and polymer modified asphalt (based on SBS) from department of highways (Thailand).

No.	Properties	Unit	**No. DH-S 401/2531*** Asphalt cement (grade AC60/70)		**No. DH-S 409/2556**** Natural rubber modified asphalt (NRMA)		**No. DH-S 408/2536***** Polymer modified asphalt (PMA) based on SBS		Test method
			Min	Max	Min	Max	Min	Max	
1	Penetration at 25°C, 100 g, 5 sec	0.1 mm	60	70	50	70	55	70	DH-T 403 ASTM D5
2	Softening point Ring and ball	°C	44	55	50		70		ASTM D36
3	Flash point	°C	232		220		220		ASTM D92
4	Penetration Index						3		NLT-181
5	Ductility at 25°C, 5 cm/min	cm	100						ASTM D113 DH-T 405
	Ductility at 13°C, 5 cm/min	cm	-				55		
6	Torsional Recovery at 25°C	%					70		NLT-329
7	Elastic recovery at 25°C	%	-		40		70		
8	Float test at 60°C	sec					3000		ASTM D139
9	Toughness / Tenacity at 25°C								ESM NE-31
	-Toughness	kg cm					170/200		
	-Tenacity	kg cm					100		

10	Brookfield viscosity (Shear rate of 18.6 s^{-1}, spindle 21 type)								ASTM D4402
	-135°C						1100		
	-150°C				200	600			
	-165°C						300		
11	Storage stability at 163-165°C, 24/120 hours difference in softening point	°C			4 (24 hrs)		2 (120 hrs)		NLT-328 ASTM D7173
12	Solubility in trichloroethylene (TCE)	%	99						DH-T 409
13	Density at 25°C	g/cm^3					1.00	1.05	ASTM D70
14	$G^*/\sin\delta > 1$ kPa at	°C			70		76.00		D7175

*http://www.doh.go.th/doh/images/aboutus/standard/03/dhsp401-31.pdf

**http://www.doh.go.th/doh/images/aboutus/standard/03/dhsp409-56.pdf

***http://www.doh.go.th/doh/images/aboutus/standard/03/dhsp408-36.pdf

Evaluation of gel content and swelling ratio by Solvent Extraction Technique

To evaluate the degree of crosslinking in terms of gel content and swelling ratio of the gel, the solvent extraction method was used. During the extraction, solvent (i.e., hot xylene) was diffused into the free volume between the crosslinking points, leading to the swelling behavior of the polymer. The non-crosslinked fraction was extracted from the blends, and the highly crosslinked fraction (gel) remained. This technique was performed according to ASTM D2765 [2].

The polymer sample was cut into small pieces which the size was less than 2 mm. It was weighed as W_0 before packed in the mesh bag and was weighed as W_1. It was then immersed in the boiled xylene (about 140 °C) for 8 hours in order to remove the soluble part of the polymer. The remained sample in mesh bag which was swollen was weighed as W_2. Eventually, it was vacuum dried at 80 °C for 6 hours to remove the xylene residue. Finally, the sample was weighed as W_3. The gel content and swelling ratio of gel were calculated using the following equations:

$$Gel\ content\ (\%) = \frac{W_3 - (W_1 - W_0)}{W_0} \times 100$$

$$Swelling\ ratio\ of\ gel\ (\%) = \frac{W_2 - (W_1 - W_0)}{W_3 - (W_1 - W_0)} \times 100$$

where W_0 = initial sample weight (g)

W_1 = initial sample weight + bag (g)

W_2 = swollen sample weight + bag (g)

W_3 = dried sample weight + bag (g)

Calculation of fail temperature (continuous grading temperature) followed ASTM D7643

In this work, the fail temparature was calculated as continuous grading temperature using the equation as follows:

$$T_c = (\frac{log_{10}(P_s) - log_{10}(P_1)}{log_{10}(P_2) - log_{10}(P_1)}) \times (T_2 - T_1)$$

Where T_c = Continuous grading temperature

P_s = Specification requirement for property in question,
for performance grading of original binder, $P_s = \left(\frac{|G^*|}{\sin\delta}\right)$ = 1 kPa

$P_2 = \left(\frac{|G^*|}{\sin\delta}\right)$ at T_2

$P_1 = \left(\frac{|G^*|}{\sin\delta}\right)$ at T_1

T_2 = Upper bound of test temperature

T_1 = Lower bound of test temperature

The equation becomes as follows:

$$T_c = (\frac{log_{10}(1) - log_{10}\left(\left(\frac{|G^*|}{\sin\delta}\right) at\ T_1\right)}{log_{10}\left(\left(\frac{|G^*|}{\sin\delta}\right) at\ T_2\right) - log_{10}\left(\left(\frac{|G^*|}{\sin\delta}\right) at\ T_1\right)}) \times (T_2 - T_1)$$

Neat asphalt;

T_2 = 70 °C, T_1 = 64 °C, $P_2 = \left(\frac{|G^*|}{\sin\delta}\right)$ at T_2 = 0.6 kPa, $P_1 = \left(\frac{|G^*|}{\sin\delta}\right)$ at T_1 = 1.3 kPa

$$T_c = \left(\frac{log_{10}(1) - log_{10}(1.3)}{log_{10}(0.6) - log_{10}(1.3)}\right) \times (70 - 64) = 66.0\ ℃$$

PMAD0;

T_2 = 76 °C, T_1 = 70 °C, $P_2 = \left(\frac{|G^*|}{\sin\delta}\right)$ at T_2 = 0.7 kPa, $P_1 = \left(\frac{|G^*|}{\sin\delta}\right)$ at T_1 = 1.5 kPa

$$T_c = \left(\frac{log_{10}(1) - log_{10}(1.5)}{log_{10}(0.7) - log_{10}(1.5)}\right) \times (76 - 70) = 73.2\ ℃$$

PMAD0.5;

T_2 = 76 °C, T_1 = 70 °C, $P_2 = \left(\frac{|G^*|}{\sin\delta}\right)$ at T_2 = 0.7 kPa, $P_1 = \left(\frac{|G^*|}{\sin\delta}\right)$ at T_1 = 1.5 kPa

$$T_c = \left(\frac{log_{10}(1) - log_{10}(1.5)}{log_{10}(0.7) - log_{10}(1.5)}\right) \times (76 - 70) = 73.4\ ℃$$

PMAD1;

$T_2 = 76$ °C, $T_1 = 70$ °C, $P_2 = \left(\frac{|G^*|}{\sin\delta}\right)$ at $T_2 = 0.7$ kPa, $P_1 = \left(\frac{|G^*|}{\sin\delta}\right)$ at $T_1 = 1.6$ kPa

$$T_c = \left(\frac{log_{10}(1) - log_{10}(1.6)}{log_{10}(0.7) - log_{10}(1.6)}\right) \times (76 - 70) = 73.6\ ℃$$

PMAD1.5;

$T_2 = 76$ °C, $T_1 = 70$ °C, $P_2 = \left(\frac{|G^*|}{\sin\delta}\right)$ at $T_2 = 0.8$ kPa, $P_1 = \left(\frac{|G^*|}{\sin\delta}\right)$ at $T_1 = 1.6$ kPa

$$T_c = \left(\frac{log_{10}(1) - log_{10}(1.6)}{log_{10}(0.8) - log_{10}(1.6)}\right) \times (76 - 70) = 73.7\ ℃$$

The fail temperatures of each sample are summarized in Table 3 of the manuscript.

**Sample name**	$\left(\frac{\lvert G^*\rvert}{\sin\delta}\right)$ **(kPa)**				**Fail temperature (°C)**
	**58°C**	**64°C**	**70°C**	**76°C**	
Neat asphalt	3.1	1.3	0.6	-	66.0
PMAD0	-	3.0	1.5	0.7	73.2
PMAD0.5	-	3.1	1.5	0.7	73.4
PMAD1	-	3.2	1.6	0.7	73.6
PMAD1.5	-	3.3	1.6	0.8	73.7

References

1. Kunanusont, N.; Samthong, C.; Bowen, F.; Yamaguchi, M.; Somwangthanaroj, A. Effect of Mixing Method on Properties of Ethylene Vinyl Acetate Copolymer/Natural Rubber Thermoplastic Vulcanizates. *Polymers* **2020**, *12*, 1739.
2. ASTM International. Standard Test Methods for Determination of Gel Content and Swell Ratio of Crosslinked Ethylene Plastics. ASTM International: West Conshohocken, PA, 2001; Vol. ASTM D2765 - 01.
